# Starch sources influence lipidaemia of diabetic dogs

**DOI:** 10.1186/s12917-019-2224-y

**Published:** 2020-01-03

**Authors:** Fabio Alves Teixeira, Daniela Pedrosa Machado, Juliana Toloi Jeremias, Mariana Ramos Queiroz, Cristiana Ferreira Fonseca Pontieri, Marcio Antonio Brunetto

**Affiliations:** 1 School of Veterinary Medicine and Animal Science, University of São Paulo, São Paulo/Pirassununga, Av. Prof. Orlando Marques de Paiva, 87 - Butantã, São Paulo, 05508-010 Brazil; 2 Grandfood Industria e Comercio LTDA, km 204, Dourado, São Paulo, 13590-000 Brazil; 3 Department of Animal Nutrition and Production, School of Veterinary Medicine and Animal Science, São Paulo University, Av. Duque de Caxias Norte, 255, Pirassununga, SP Brazil

**Keywords:** Cholesterol, Triglycerides, Nutrition, Endocrinopathy

## Abstract

**Background:**

Hyperlipidaemia is considered a cause of other diseases that are clinically important and potentially life threatening. Combination of pea and barley as exclusive starch sources is known to interfere with glycemic control in diabetic dogs, but their effect on lipid profile of hiperlipidaemic dogs is yet to be evaluated. Twelve adult diabetic dogs were fed three dry extruded diets with different starch sources and different fat levels: peas and barley (PB), maize (Mi), and peas, barley and rice (Ba) with 15.7, 15.6 and 9.0% of their dry matter as fat, respectively. Plasmatic cholesterol and triglycerides concentration curves over 10 h were obtained after 60 days on each diet and with the same NPH insulin dose. ANOVA test or Friedman test were used to compare the dietary effects on triglycerides and cholesterol variables among the diets.

**Results:**

Dogs presented lower mean (*p* = 0.05), fasting (*p* = 0.03), and time 8-h postprandial (p = 0.05) triglyceridemia after PB diet period than Ba diet period and time 4-h postprandial (*p* = 0.02) lower after PB than Mi diet. Cholesterolemia mean, minimum, maximum, area under the cholesterol curve and times points: 2, 4, 6, 8 and 10-h postprandial, had lower values after PB ingestion in comparison to Mi, without difference to Ba diet.

**Conclusion:**

Inclusion of pea and barley, as exclusive starch sources, in therapeutic diets for diabetic dogs can minimize plasmatic triglycerides and cholesterol concentration at fasting and at different postprandial time, compared to the maize diet or diet with lesser fat content.

## Background

Hyperlipidaemia refers to an increased concentration of lipids in the blood. It is associated with other canine diseases that are clinically important and potentially life threatening such as pancreatitis [1–3], gall bladder mucocele [4], atherosclerosis [5–9], ocular [10] and neurologic diseases [11, 12]. Specifically about Miniature Schnauzers, hyperlipidemia was associated to hepatopathies [13], insulin resistance [14] and proteinuria [15, 16].

Secondary hyperlipidaemia is the most common form of hyperlipidaemia in dogs [17], mainly resulting from an endocrine disorder [18–20], such as diabetes mellitus (DM). Thereupon, it has been recommended that diets for diabetic dogs must be moderate to low in fat [21], since it may minimize plasmatic lipid concentrations; and high fiber content since it may lead to a decrease in blood glucose levels [22–25]. However, there is evidence suggesting that the low-fat and high-fiber combination has side effects such as deficient weight gain, bulk and softened feces, flatulence, constipation, vomit, opaque hair and lesser palatability [21–26], probably due to the effect of dietary fiber on dog microbiota [27]. Therefore, new strategies should be investigated to improve diet design for diabetic dogs.

Starch source affect significantly the digestibility of the diet [28]; and interferes with the postprandial glycemic curves in diabetic dogs [29, 30]. Previously, our group showed that pea and barley mix as exclusive dietetic starch minimize postprandial glycemia in diabetic dogs compared to maize [29]. But studies focusing on lipid profile of dogs with endocrinopathies feeding different starch sources were not found.

Thus, this study aims to assess the influence of two levels of dietary fat content, and two starch sources – pea with barley vs maize – on the lipid profile of dogs with stable DM.

## Results

**Fig. 1:**
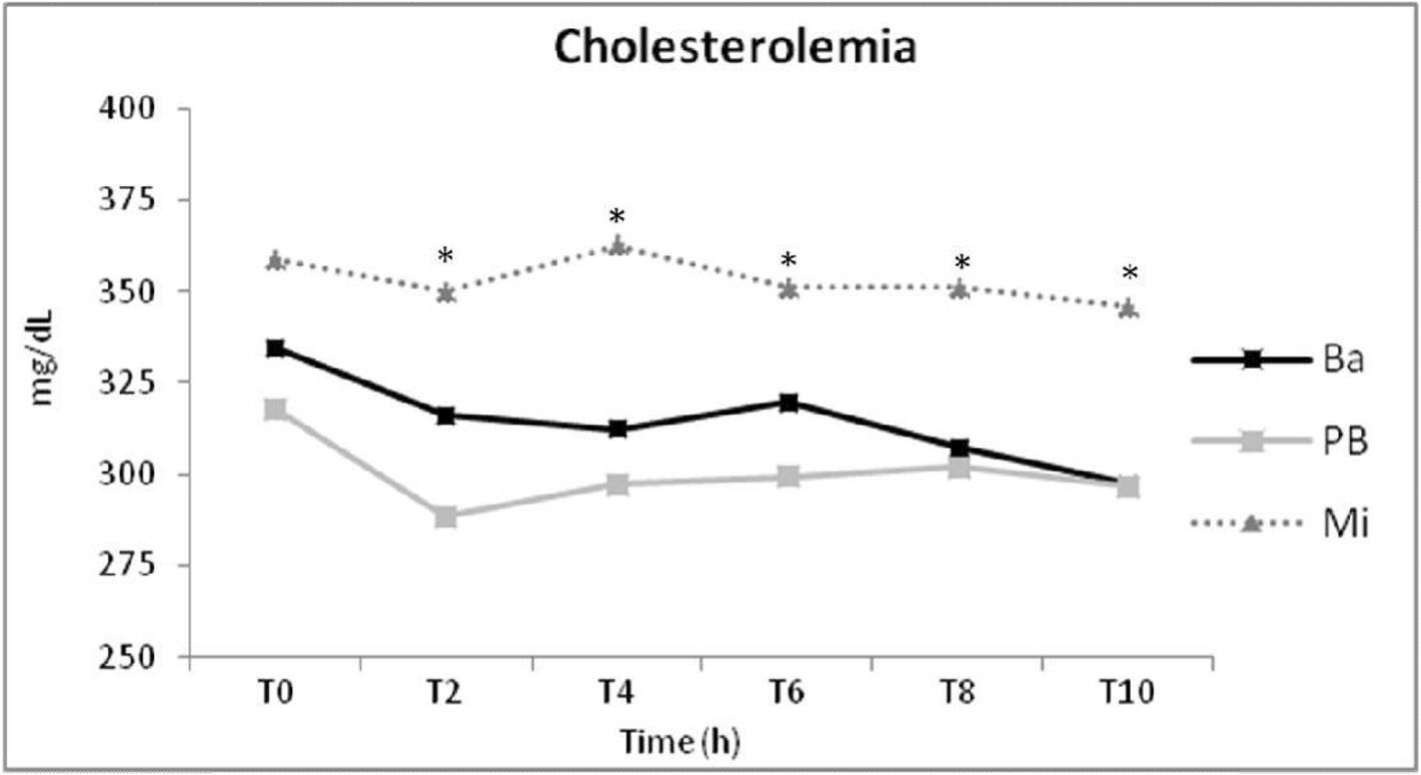
Mean plasmatic triglycerides concentration of 12 diabetic dogs after 2 months feeding of basal (Ba), pea with barley (PB) and maize (Mi) diets. *Time with difference among diets

**Fig. 2:**
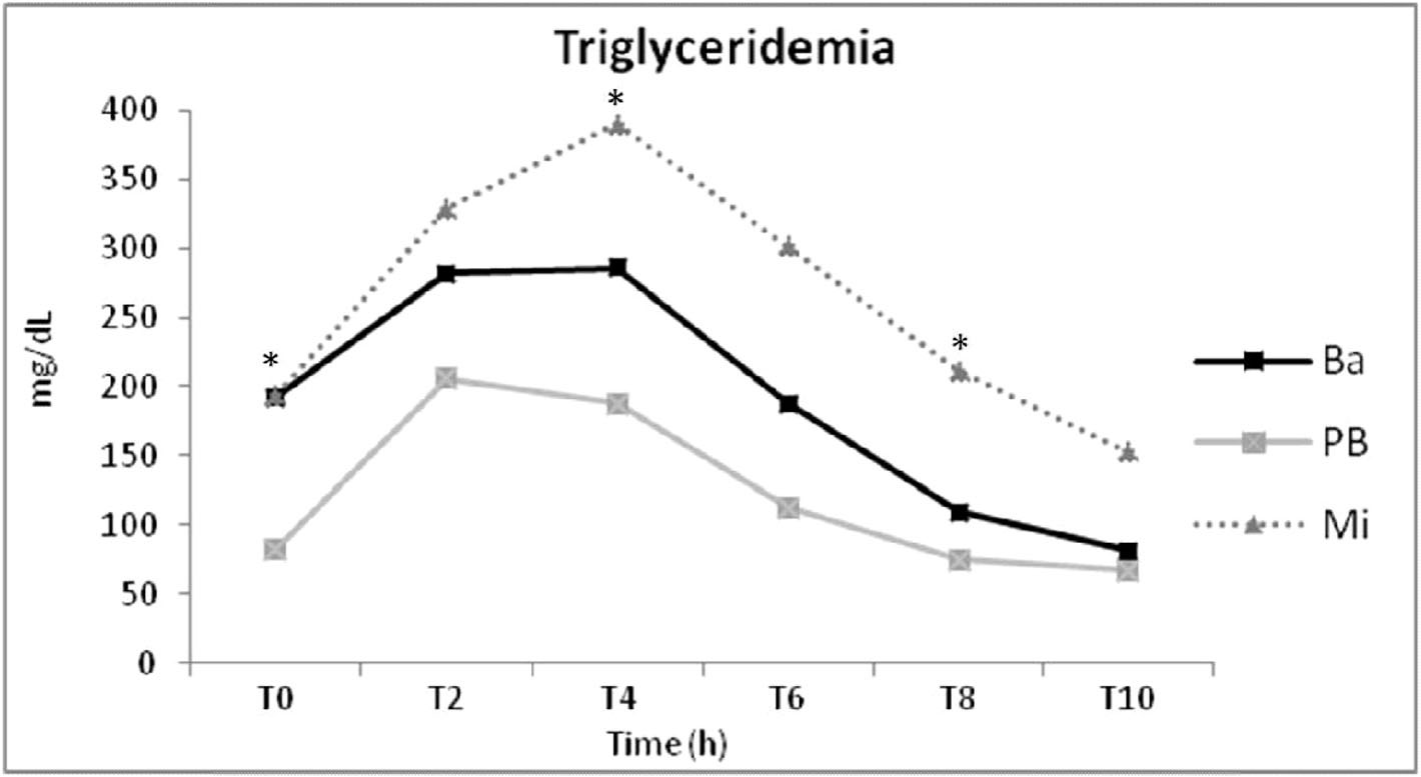
Mean plasmatic triglycerides concentration of 12 diabetic dogs after 2 months feeding of basal (Ba), pea with barley (PB) and maize (Mi) diets. *Time with difference among diets

After consumption of each diet for 2 months, and under the same insulin dosage; the lipid metabolism of diabetic dogs was evaluated by 10-h plasmatic triglycerides (Fig. 1) and cholesterol (Fig. 2) concentration curves. Twelve animals were included in the analysis (Table 1). Patients ranged from 5 to 12 years of age with a mean of 8.3 years. No significant variation (*p* = 0.12) of body weight were observed at the end of each diet administration period (Table 1).

**Table 1:** Characteristics of 12 diabetic dogs at the beginning of the study

Breed	Sex	Age (years)	Body weight (kg)^a^			Body condition score	NPH insulin (unit/kg)	
			Post-Ba	Post-PB	Post-Mi		Morning	Night
Mixed breed	Intact male	7	10.5	10.4	10.0	4	0.67	0.67
Labrador retriever	Neutered male	9	30.1	31.5	31.4	5	0.50	0.50
Mixed breed	Spayed female	9	5.8	5.7	6.0	5	0.17	0.17
Cocker	Spayed female	5	12.5	12.9	12.1	5	0.64	0.64
Mixed breed	Spayed female	12	10.4	10.4	10.4	5	0.19	0.19
Labrador retriever	Spayed female	8	29.5	30.3	30.4	5	0.34	0.34
Labrador retriever	Intact male	7	45.5	45.0	45.6	5	0.40	0.40
Schnauzer	Neutered male	7	8.9	8.5	8.5	4	0.68	0.68
Labrador retriever	Intact male	7	38.0	38.6	38.0	5	0.39	0.39
Labrador retriever	Spayed female	10	25.1	26.4	26.3	5	0.40	0.40
Pug	Spayed female	9	9.5	10.0	10.0	6	0.85	0.85
Dachshund	Spayed female	9	6.6	6.7	6.6	5	0.23	0.23

^a^Without difference after each diet period (*p* = 0.12 obtained by ANOVA test)

The means from the variables obtained from the triglycerides and cholesterol curves for each animal after each dietary treatment were compared (Table 2).

**Table 2:** Mean and standard deviation of plasmatic triglycerides and cholesterol concentration of 12 diabetic dogs after 2 months feeding of basal (Ba), pea with barley (PB) and maize (Mi) diets

Variables (mg/dL)	Triglycerides							Cholesterol						
	Ba	sd	PB	Sd	Mi	sd	*P*	Ba	sd	PB	sd	Mi	sd	*P*
T0	192.4^†^	238.8	83.8^‡^	43.9	194.8^†‡^	283.3	0.03^**^	334.6	113.3	318.1	96.7	358.8	119.6	0.11^*^
T2	282.1	262.8	205.9	144.4	328.4	340.2	0.44^**^	316.4^†,‡^	100.1	288.7^†^	85.2	350.2^‡^	113.9	0.02^*^
T4	285.9^†‡^	254.7	188.3^†^	175.9	389.5^‡^	392.9	0.02^**^	312.5^†^	106.0	297.2^†^	101.4	362.7^‡^	127.1	0.01^*^
T6	187.6	167.5	113.4	125.3	301.2	381.4	0.10^**^	319.9^†‡^	106.3	299.5^†^	102.5	351.2^‡^	112.3	0.02^*^
T8	109.9^†^	76.7	75.8^‡^	46.6	211.6^†‡^	306.6	0.05^**^	307.6^†‡^	123.8	302.1^†^	91.6	351.0^‡^	117.1	0.03^*^
T10	81.5	51.0	67.5	30.2	152.7	195.5	0.37^**^	297.6^†^	113.9	296.7^†^	93.0	345.9^‡^	115.3	0.02^*^
Mean	189.9^†^	161.9	120.6^‡^	79.1	263.0^†‡^	309.9	0.05^**^	314.8^†‡^	108.6	303.0^†^	96.3	353.3^‡^	116.6	0.02^*^
Minimum	76.4	51.7	63.7	30.7	141.9	198.8	0.34^**^	282.7^†^	104.4	284.5^†^	85.2	334.2^‡^	108.5	<0.01^*^
Maximum	316.8	260.4	221.7	180.5	397.8	386.3	0.10^*^	341.4^†‡^	116.5	323.9^†^	102.7	376.2^‡^	122.6	0.02^*^
Δ	240.5	244.3	158.0	175.9	255.9	224.5	0.18^*^	58.7	37.6	39.4	22.9	42.0	24.2	0.18^*^
AUC	2004.7	1692.0	1349.8	919.5	2809.0	3256.1	0.09^**^	3145.2^†^	1081.6	2856.0^†^	819.9	3534.8^‡^	1166.9	0.01^*^
AUIC	81.1	925.4	509.7	582.6	861.2	1078.5	0.12^*^	−201.2	238.3	− 183.0	141.9	−52.8	175.7	0.20^*^

*sd* Standard deviation, *AUC* Area under the curve, *AUIC* Area under the increment curve; Δ, difference between maximum and minimum values

^†,‡^ Different superscript symbols means statistically difference in line between diets (*p* < 0.05)

^*^P value obtained by ANOVA test (post-hoc Tukey test)

^**^P value obtained by Friedman (post-hoc multiple comparisons test)

Regarding plasmatic triglycerides concentration, mean (*p* = 0.05), fasting (t0; *p* = 0.03), and time 8-h postprandial (t8; *p* = 0.05) were lower after PB diet period than Ba diet. At time 4-h postprandial (t4; *p* = 0.02), PB diet resulted in lower plasmatic triglycerides levels than Mi diet (Table 2).

Cholesterol differed more than triglyceridemia concerning the two isonutrient diets. Only fasting (t0), difference between maximum and minimum, and AUICC presented no difference between diets periods (Table 2). All difference variables (*p* < 0.05; Table 2) showed higher cholesterol concentrations after Mi diet period than PB diet, similar values between PB and Ba diet periods, and some variables (t2, t6, t8, mean, maximum) did not differ between the Mi and Ba diet (Table 2).

## Discussion

This clinical trial results showed a possible lipid-lowering effect of PB diet. This influence of PB diet on plasmatic cholesterol and triglycerides concentrations could be attributed to the difference of dietary fat levels. Indeed, the only publication, to our knowledge, that evaluated the amount of dietetic fat for diabetic dogs, also showed a decrease in lipid profiles in response to lower dietary fat levels [21]. Although, in our study comparing PB and Mi, they both had the same ethereal extract content (Table 3), so dietetic fat amount did not justify the lipid lowering effect of PB diet. Moreover, differently from Fleeman et al. [21], which studied diabetic dogs, and similar to healthy dog evaluated by Elliot et al. [31], our results showed more similarities in plasmatic lipid concentrations than differences among the lowest fat diet (9.0% in dry matter basis) and the others diets (around 15.6%). Probably, Fleeman et al. [21] observed significant influence of dietary fat content on plasmatic lipid concentration because they had a greater difference in the amount of dietary fat being compared (from 2.45 to 5.75 g of fat /100 kcal) than this study (2.78 to 4.09 g of fat /100 kcal) and that of Elliot et al. [31] [2.70 to 3.96 – values determined using modified Atwater factors [32] based on information of percentage of metabolizable energy from fat, expressed by authors]. Thus, we suggest that there are other factors involved in the hypolipidemic effect of the PB diet in our observations.

**Table 3:** Chemical composition of basal (Ba), pea with barley (PB) and maize (Mi) diets

Item (% dry matter)	Ba^a^	PB^b^	Mi^c^
Crude protein	39.5	37.2	34.7
Ethereal extract (hydrolysis)	9.0	15.7	15.6
Total dietary fiber	19.6	20.6	19.3
Soluble fiber	1.6	3.3	1.0
Insoluble fiber	18.0	17.3	19.3
Ash	6.3	6.3	5.6
Starch	19.1	19.7	21.4
Metabolizable energy (kcal/g DM)	3.2	3.8	3.9

^a^chicken by-product meal, wheat gluten, swine isolate protein, pea flour, barley, brewer’s rice, porcine plasma powder, dried egg, cellulose, beet pulp, poultry fat, fish oil, β-glucan, gelatin hydrolisate

^b^Pea flour, barley, chicken by-product meal (24.0%), wheat gluten, pork fat (2.0%), swine isolate protein, cellulose, beet pulp, poultry fat (6.0%), fish oil

^c^Maize, chicken by-product meal (32.0%), wheat gluten, pork fat (2.0%), swine isolate protein, cellulose, beet pulp, chicken fat (4.3%), fish oil

Regarding the ingredients, PB and Mi have the same fat sources with small differences in the amount included. PB diet has the same percentage of fish oil and pork fat included, higher poultry fat (6.0 X 4.3%), and lower chicken by-product inclusion (24.0 X 32.0%) than Mi diet (Table 3). Considering that chicken by-product has around 14.0% fat, the inclusion of this ingredient in the Mi diet would not result in higher poultry fat amount than PB diet. Moreover, it has been shown that dietary fat source influences serum cholesterol concentrations in dogs, irrespective of dietary protein source [33]. Therefore, it was expected that PB would translate in higher cholesterolemia than Mi diet because of the greater poultry fat inclusion; however, fat source does not explain the lower plasmatic lipid concentration after the PB diet period.

In turn, dietary starch amounts could alter postprandial glucose response in healthy pets [34, 35] and lipid response in humans [36], but the three diets had close starch amounts.

A better explanation would be the possibility that peas and barley act as functional foods for diabetic dogs due to the possible presence of components that can minimize hyperlipidemia. An early study comparing the effects of peas or maize (corn flakes) addition in the feed of humans with different degrees of cholesterolemia, observed a decrease in the concentration of cholesterol after consumption of pea [37]. The authors speculated that higher consumption of pea fiber was the cause of these effects. Since it was more fermentable it would promote higher excretion of bile acids due to direct effect on transit time and the fiber’s direct stimulation would increase the content of bile acids in the feces.

Furthermore, barley is known to contain an abundance of soluble and insoluble fiber. Studies in humans and rat models, show that barley’s fiber content is the main feature associated to better lipid indexes, predominantly soluble fiber [38–41]. In our study, despite the similarity into the total dietary fiber content among the three diets used, the PB diet had a higher amount of soluble fiber, which could justify part of its positive effect on plasma lipid concentration. Indeed, previous studies with diabetic dogs showed lower fasting cholesterolemia after ingestion of insoluble high fiber diet [23] and undistinguished high fiber diet [22].

A systematic review [41] attributed the use of barley to significantly lower total cholesterol, low-density lipoprotein cholesterol and triglycerides in humans due to high content of β-glucan inherent to this ingredient. β-glucan is also associated to reduced gastric emptying, digestion, and absorption of cholesterol and fat, and increased excretion of bile acids, neutral sterols and catabolism of cholesterol [38, 39]. Despite the lack of a direct analysis of dietary β-glucan content, the inclusion of β-glucan in the commercial Ba diet (Table 3) and the presence of barley at PB diet could explain some similarities in the results regarding triglyceridemia and cholesterolemia between these diets, as well as the differences to Mi diet.

Recent studies also showed that pea protein can result in decrease of blood triglycerides and cholesterol concentration in different species [37, 42–45]. Possible mechanisms of action are not fully clarified but researchers correlated the consumption of pea protein to high hepatic LDL-receptor mRNA concentration, consequently increasing hepatic LDL receptor leading to accelerated clearance of LDL-cholesterol particles [42]; along with lower fatty acid synthesis due to down regulation of hepatic mRNA concentrations of fatty acid synthase and stearoyl-CoA desaturase – fatty acids synthetizing enzymes [42]; and reduced hepatic cholesterol concentration due to higher excretion of bile acids via feces, an effect which is probably mediated by an up-regulation of the enzymes of bile acid synthesis [43, 44]. It is debated whether different levels of amino acids or specified pea peptides are responsible for these lipid-lowering effects. Further studies are necessary to isolate the compound responsible for this effect on lipids blood level in diabetic dogs.

In addition to the possible effects intrinsic to pea and barley compounds making them functional foods, there is still the possibility that the lipid-lowering effect of pea and barley is associated to the diet’s glycemic index. A recent metabolomic study with obese humans found differential regulation of certain amino acids and phospholipids depending on the diet’s glycemic index [46], which could contribute to explain the lipid-lowering effects attributed to low-glycemic index diet on diabetic or hyperlipidemic humans reported previously [47, 48]. It was not measured in our study, but we have already shown that a pea and barley-based diet generate lower glycemic variables in diabetic dogs when compared to a maize-based diet [29]. Additionally, Adolphe et al. [49] pointed that pea has lower glycemic index compared to others starch sources as rice in dogs.

Thus, since peas and barley appear to minimize triglyceridemia and cholesterolemia in diabetic dogs, we believe they should be considered functional foods to dogs with endocrinopathies. Although, it is still a subject of controversy which components of these ingredients are responsible for their effects on lipid metabolism.

### Particularities and limitations

First, an important factor in this research is the isolation of the effect of the diet on blood lipid concentration, because lipid measurements were performed in stable diabetic dogs since the amount of insulin administered was kept constant. Insulin is known to act on several metabolic pathways, including lipid metabolism and there are evidences that high blood triglycerides concentration is associated with insulin resistance [14, 19]. We did not find previous studies on the influence of insulin type in blood lipid concentration, thus all dogs in this study received NPH-type insulin to homogenize factors influencing the lipid metabolism. In addition, there was no variation at animals’ body weight. This is important for studies on hyperlipidemia, due to association between high BCS and increases in concentration of triglycerides and cholesterol [50]. Only one dog had BCS higher than ideal (6/9). That excess of adipose tissue probably was not sufficient to cause significant metabolic changes and that dog keept the same BCS (6/9) during all study period.

Second, the methodology chosen to evaluate effect of diet on hyperlipidemia of diabetic dogs was: plasmatic triglycerides and cholesterol concentration measurement by a curve during 10 h, every 2 hours. Some studies that evaluated dietary effects on blood triglycerides and cholesterol concentration used only fasting blood samples to measure these lipid’s concentrations [22, 23], but it was observed that fasting triglyceride concentrations do not predict the highest postprandial triglyceridemia in healthy dogs [31, 51]. Higher differences at plasmatic triglycerides concentrations at different times than fasting was observed in our data and in other studies that evaluated triglyceridemia and cholesterolemia of diabetic dogs by the similar methods to ours [21]. Moreover, in our study, fasting cholesterolemia did not show significant difference between diets, but all other times of curve did. Elliot et al. [31] considered these findings relevant to diabetic dogs because they are fed and given exogenous insulin therapy every 12 h.

Although 12 animals represent a small sample size, based on previous research this study included the similar number of diabetic dogs evaluated for dietary modifications under a crossover design as other studies [21–23, 29]. All the animals were spontaneous diabetic dogs and were included after a rigorous selection, considering even the owners commitment to following the protocol. Therefore, we were able to keep the animals under a strict diet, avoiding differences in food intake which could have interfered in lipid metabolism. We did not find another published research that compared isonutrients diets focusing on lipids concentration in diabetic dogs. Also, despite the lipid-lowering effect of peas and barley already being reported in other species, this is the first study to assess it in diabetic dogs.

In our data, we found a high variations (expressed as standard deviation) of the triglycerides concentration. It was observed in other published studies [52], including with diabetic dogs [21]. De Marco et al. (2017) [52] found a range of triglycerides concentration: 350 to 4356 mg/dL. The cited authors did not explain this high variation and we have no explanation as to why values vary so much between individuals. However, we believe this is due to different individual responses related to physiological absence of insulin and the individual changes that occur with other hormones such as leptin and inflammatory markers in diabetic dogs [53, 54], which need to be further studied in veterinary medicine.

An important limitation is that we cannot distinguish whether the beneficial hypolipidemic effect was due to the association of pea and barley or if they have these effects in isolation. Not all the dietary lipid classes were measured, so we cannot reject the possibility of alternative modulation by other means than pea and barley. In this context, another limitation is the fact that Ba and PB are commercial diets, which made it difficult for us to access the amount included of all the ingredients in each formulation. This also diminishes our ability to assess whether the inclusion of rice in the Ba diet had any influence on the results, because it was expected that the less fat diet, that also has peas and barley in its composition, would generate even lower blood lipids concentrations. Likewise, the diets were formulated with different fat sources and the differences in fatty acid profile could impact the lipid metabolism [55]. Probably in a further study the evaluation of all the lipid classes could clarify also this aspect.

The results of the present study suggest a hypocholesterolemic and hypotriglyceridemic effect by the association of pea and barley in diabetic dog’s diets. Our results expand the alternatives for nutritional treatment of diabetic dogs and hyperlipidemic dogs, including the possibility of an increase in the amount of dietary fat, which may be an interesting alternative for more selective dogs or for patients with a tendency to lose weight when receiving high-fiber and low-fat diets [21].

Furthermore, dogs may serve as models for human medicine due to metabolomic similarities between diabetic dogs and type-1 human diabetes mellitus [56]. The study of diabetic dogs offers better standardization of diet and treatment while experimenting on individuals with naturally acquired diabetes mellitus, instead of induced diabetes, as is commonly seen. As a result, we believe in the potential these ingredients have for positively affecting human health as well.

## Conclusions

The results obtained from this group of 12 animals under the experimental design conducted, support the claim that peas and barley included in the diet of diabetics dogs can minimize the plasmatic concentration of triglycerides and cholesterol better than maize-based diets and in a similar way to low-fat diet.

## Methods

### Animals

The animals used in this research were selected from the routine practice of the veterinary hospital of the School of Veterinary Medicine and Animal Science of the University of São Paulo as our previous study [29]. In which the medical records of 368 diabetic dogs were analyzed and 18 animals were selected according to the following exclusion criteria (intact female dogs; dogs younger than 1 year old; diagnosis of concomitant diseases; body condition score (BCS) lower than 4 or higher than 6 on a scale [57] of 1 to 9). In the current study, we added the exclusion criteria: treatment with hypolipidemic drug and/or omega-3 polyunsaturated fatty acid for less than 6 months. Sixteen diabetic dogs were included in the study, but four were excluded (one due to personal difficulties of the owner in conducting the experimental protocol, and the other three animals did not accept food in the veterinary hospital environment). Thus, 12 dogs with spontaneous DM and hyperlipidaemia historic were included in this study (Table 1). From diagnosis to start of study, for these 12 dogs the duration of DM ranged from 150 to 1034 days. All animals received Neutral Protamine Hagedorn (NPH) insulin. After the study, all animals were kept in its usual environment home with their owners.

The minimum number of animals, to achieve 80% of power calculation, was performed by glycemic values from Teshima et al. [30]. This sample size was calculated using statistical program Action® to previous research project [29] and resulted in 10 diabetic dogs.

### Diets and experimental design

**Fig. 3:**
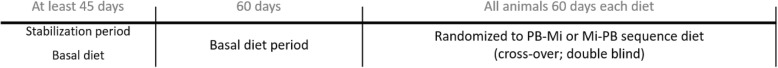
Schematic representation of study design. PB = pea and barley diet; Mi = maize diet

Dogs entered the experimental protocol after they were considered DM stabilized: at least 45 days without changes in insulin dosage due to absent manifestations of polyuria and polydipsia and blood glucose levels ranging from 5.0 to 16.7 mMol/L (90 to 300 mg/dL) [58]. During this stabilization period, all dogs had been eating the same basal (Ba) commercial dry extruded diet recommended for obese dogs^1^ (Table 3). After being considered stable, all animals received Ba diet for additional 60 days. Then, all animals were randomized to receive for 60 days two other diets (PB and Mi), in a crossover manner, via a draw of the possible PB-Mi or Mi-PB sequences (Fig. 3). PB is a commercial diet recommended for diabetic dogs^2^ with pea and barley as exclusive starch source. Mi is an experimental diet produced as isonutrient diet in relation to PB diet (Table 3), with the maize as single starch source. Animals were fed in their homes by their owner twice-a-day following NPH insulin administration, with an exact 12-h interval between meals. Insulin dose was not changed over the experimental protocol. Initial caloric requirement was estimated by maintenance formula [397 kJ x BW^0.75^ (BW = body weight in kg)] [32]. To guarantee that the study was double-blinded, owners received diets only identified with the letters A and B, and the researchers responsible for the analysis of the results and for providing the diet to the owners were unaware of the meaning of this identification letters. During experimental protocol, dogs were evaluated every 3 weeks at veterinary hospital (body weight, BCS, blood glucose concentration, and owners were asked about insulin and dietary management, and occurrence of DM manifestation). At each re-evaluation, diet was supplied to owners in enough amount for the dogs to show no change in weight (lesser than 5%).

At the end of the 60 days consuming each of the three diets (Ba, A and B), the diabetic dogs returned to the veterinary hospital in the morning post 12-h fasting conditions for plasmatic triglycerides and cholesterol curves. A blood sample was collected before feeding (fasting sample, time 0; t0). After this collection, dogs were fed, received the regular dose of insulin, and new blood samples were collected at 2 (t2), 4 (t4), 6 (t6), 8 (t8) and 10 h (t10) after feeding.

### Laboratory analysis

Blood samples were collected in tubes with fluoride EDTA. Plasma was separated after centrifugation at 3500 rpm for 5 min and kept frozen at −20 °C until analysis. Plasmatic triglycerides concentrations were analyzed by the glycerol phosphate method and plasmatic cholesterol concentration by the enzimatic oxydase/peroxidase method, both using a commercial kit.^3^ Information about diet analyses methods were described previously [29].

### Calculations and statistical analysis

In order to compare the effects of each dietary treatment on lipidemia, the 10 h triglycerides and cholesterol curves of the 12 dogs were combined and averaged. Consequently the mean triglycerides and cholesterol value for each time point (t0, t2, t4, t6, t8 and t10) and the mean values of fasting, mean, maximum, minimum and fluctuation of triglycerides and cholesterol were calculated between all dogs for each dietary treatment.

Fasting triglyceridemia and cholesterolemia were considered the plasmatic concentration at time t0 (immediately before feeding). Mean triglycerides and cholesterol were defined as the average of mean of each time point at 10-h curves values, for each dietary treatment. The maximum and minimum triglycerides and cholesterol values were defined respectively as the mean of the highest and lowest values obtained for each 10-h curves. The difference between maximum and minimum values was defined by subtracting the highest and lowest values of each curve and calculating the average of these differences. The triglycerides and cholesterol increment was calculated by normalizing measurements by subtracting t0 values for each animal. The area under the triglycerides (AUTC) and cholesterol (AUCC) curves and the area under the increment curve (AUTIC and AUCIC) were calculated via numerical integration using the trapezoidal method.

Statistical analyses and numerical integration using the trapezoidal method were performed in a statistical software.^4^ Initially, Bartlett test was applied to verify the homogeneity of variances for triglycerides and cholesterol variables, and body weight after 60 days receiving each diet. Significant variation in all time points of the triglycerides curve, mean triglycerides, average of minimum triglycerides and AUTC, were assessed using Friedman’s test and post-hoc multiple comparisons test. One factor Anova was conducted on all cholesterol variables (fasting, t2, t4, t6, t8 and t10 curve, mean, maximum, minimum, difference between maximum and minimum, AUCC and AUCIC), to maximum triglycerides, difference between maximum and minimum triglycerides, AUTIC and body weight, due to these variables showing homogeneous variance; followed by a post-hoc Tukey test. For all tests, the α value established for significant results was 0.05 (*p* value <0.05).

## Data Availability

The datasets used and/or analysed during the current study are available from the corresponding author on reasonable request.

## Abbreviations

AUCC: Area under the cholesterol curve
AUCIC: Area under the triglycerides increment curve
AUTC: Area under the triglycerides curve
AUTIC: Area under the triglycerides increment curve
Ba: Basal diet
BCS: Body condition score
BHA: Butylated hydroxyanisole
BW: Body weight
DM: Diabetes mellitus
Mi: Maize based-diet
NPH: Neutral protamine Hagedorn
NRC: Nutrient requirements council
PB: Pea and barley based diet

## Footnotes

1 Premier Nutrição Clínica Obesidade Cães

2 Premier Nutrição Clínica Diabetes Cães

3 Biosystem S.A., Barcelona, Spain

4 R Core Team. R: A Language and Environment for Statistical Computing; R Foundation for Statistical Computing, 2016.
